# Living with knowledge gap and psychological burdens: understanding the attitudes, barriers, and support needs for pulmonary rehabilitation among oral and maxillofacial cancer patients in China

**DOI:** 10.1007/s00520-025-10301-6

**Published:** 2026-02-06

**Authors:** Jingya Yu, Lixia Kuang, Xie Yu, Yu Zhang, Xuemei Yang, Lu Bai, Liyan Mao, Xiaoqin Bi

**Affiliations:** 1 State Key Laboratory of Oral Diseases & National Center for Stomatology & National Clinical Research Center for Oral Diseases & Dep. Of Orthognathic and TMJ Surgery, West China Hospital of Stomatology, Sichuan University, Chengdu, 610041 China; 2 West China School of Nursing, Sichuan University, Chengdu, 610041 China; 3 Department of Hepatobiliary, Chongqing Fuling Hospital, School of Medicine, Chongqing University, Chongqing, 408000 China; 4 Department of Head and Neck Oncology, West China Hospital of Stomatology, Sichuan University, Chengdu, 610041 China; 5 Division of Head & Neck Tumor Multimodality Treatment, West China Hospital, Sichuan University, Cancer Center, Chengdu, 610041 China; 6 Department of Stomatology, Longhua People’s Hospital of Shenzhen, Shenzhen, Guangdong, 518109 China

**Keywords:** Head and neck cancers, Qualitative study, Pulmonary rehabilitation, Caregiver, Experience, Rehabilitation

## Abstract

**Abstract:**

**Purpose:**

This study aimed to explore the attitudes, challenges, and support needs related to pulmonary rehabilitation (PR) among patients with oral and maxillofacial cancers and their caregivers in China.

**Methods:**

We conducted semi-structured, in-depth interviews with a purposive sample of patients, both pre- and post-surgery, and their caregivers at a tertiary hospital in Chengdu, China. Interviews were audio-recorded, transcribed verbatim, and analyzed using thematic analysis.

**Results:**

A total of 14 participants (7 patients and 7 caregivers) were interviewed. Five key themes and thirteen sub-themes emerged: (1) perceptions and attitudes toward PR, (2) multifactorial influences on participation, (3) preferences for PR education formats, (4) challenges in engaging with PR, and (5) psychological burden and the need for information support.

**Conclusion:**

This study offers critical insights into the barriers and facilitators of PR for oral and maxillofacial cancer patients in China. Findings underscore the need for culturally tailored, patient-centered PR programs that address both physical and psychological recovery. Improved PR initiatives could enhance pulmonary function, quality of life, and overall outcomes for this vulnerable population, offering valuable implications for healthcare stakeholders and policymakers in China and similar settings worldwide.

**Supplementary Information:**

The online version contains supplementary material available at 10.1007/s00520-025-10301-6.

## Introduction

Oral and maxillofacial cancers, primarily classified under the ICD-10 code range C00–C14 (malignant neoplasms of lip, oral cavity, and pharynx), are a significant global health concern. While globally ranked as approximately the 16th most common malignancy and contributing substantially to the overall burden of head and neck cancers, their incidence and prevalence vary markedly across geographic regions, influenced by heterogeneous environmental exposures, lifestyle patterns, and population-specific risk factors [1–3]. The standard treatment approach typically involves multimodal therapies, including surgery, radiotherapy, and chemotherapy. Among these, complex surgical techniques such as microvascular free flap reconstruction play a critical role in restoring both function and aesthetics following tumor resection [4]. However, these surgeries are often associated with significant postoperative complications, particularly postoperative pulmonary complications (PPCs), which frequently emerge within the first 7 days after surgery and encompass a spectrum of conditions including pulmonary edema, pneumonia, pulmonary embolism, and acute respiratory distress syndrome (ARDS) [5]. PPCs affect 3% to 38% of patients and are especially common following oral cancer surgeries involving free-flap reconstruction, where factors such as prolonged anesthesia duration and the presence of a tracheostomy substantially increase risk, ultimately contributing to higher morbidity, extended hospital stays, and increased healthcare costs [6–8].

In response, pulmonary rehabilitation (PR), a comprehensive, multidisciplinary approach combining exercise, education, and behavioral strategies [9, 10], has been demonstrated to enhance respiratory function, reduce postoperative pulmonary complications, and improve overall clinical outcomes [11]. This approach has shown particular efficacy in patients with chronic obstructive pulmonary disease (COPD) [12, 13], with evidence supporting that exercise-based PR enhances exercise capacity, improves quality of life, and alleviates dyspnea in individuals with severe COPD. While PR is well-established in the management of COPD, its role in oncologic surgical populations, particularly those with head and neck malignancies, remains underexplored [14].

Currently, China is experiencing accelerated population aging, with adults aged 65 and older increasing from 7.0% in 2000 to 12.6% in 2019, and projections estimate that this group will comprise 26.3% of the population by 2050 [15]. Aging is accompanied by progressive declines in pulmonary structure and function, often exacerbated by comorbidities such as smoking-induced airway damage [16, 17]. Although oral and maxillofacial cancers have traditionally been more prevalent among older males with a history of tobacco use, who remain the most susceptible to postoperative pulmonary complications, recent epidemiological data also indicate a gradual shift, with studies reporting a modest rise in cases among younger adults [18]. The complexity of surgical interventions in this aging population, often involving extensive incisions and disruption of the upper respiratory tract, further increases the risk of swallowing dysfunction, aspiration, and respiratory infections [19]. Given these multifaceted challenges, PR represents a potentially valuable adjunct in enhancing recovery and functional outcomes in this high-risk group.

Recent healthcare reforms in China, such as the adoption of diagnosis-related group (DRG) payment systems and enhanced recovery after surgery (ERAS) protocols, have shortened hospital stays and emphasized rapid recovery [20, 21].  Although these initiatives aim to enhance healthcare efficiency, they concurrently shift greater responsibility onto patients for managing their postoperative recovery. This transition highlights the critical need for comprehensive pulmonary rehabilitation education and self-management support, particularly during the shift from hospital to home care. Despite its clinical significance, limited qualitative research has explored how patients with oral and maxillofacial cancer perceive pulmonary rehabilitation or the barriers they face in its uptake. Existing studies have predominantly emphasized clinical outcomes and physiological measures, often neglecting the lived experiences, attitudinal dynamics, and sociocultural factors that influence engagement with rehabilitation. To address this knowledge deficit, the present study employs a qualitative design to explore the experiences, barriers, and support needs related to PR among Chinese patients with oral and maxillofacial cancer and their caregivers. The inclusion of caregivers is crucial, as family members offer both practical and emotional support, which plays a pivotal role in enhancing treatment adherence, promoting a sense of normalcy, and ensuring continued access to healthcare services [22]. Additionally, understanding caregivers’ characteristics and needs enables healthcare professionals to design and implement more tailored and effective interventions. By generating contextually grounded insights, the findings aim to inform the development of culturally responsive, patient-centered pulmonary rehabilitation interventions, thereby contributing to improved rehabilitation strategies and guiding future oncologic care in the Chinese healthcare system.

## Method

### Study design

This qualitative study employed semi-structured interviews to explore the attitudes, barriers, and support needs of patients with oral and maxillofacial cancer in China. Ethical approval was granted by the Institutional Review Board (IRB) at the West China Hospital of Stomatology, Sichuan University (grant number WCHSIRB-D-2025-025). Informed consent and permission to use the audio recording device were obtained from all participants before their inclusion in the study. The informed consent process involved a thorough review of the study’s risks, benefits, and the patient’s right to withdraw at any time without consequence. The study findings were reported in accordance with the Consolidated Criteria for Reporting Qualitative Research (COREQ) guidelines [23].

### Participants

Purposive sampling was used to recruit the participants. Participants were recruited from the Department of Head and Neck Oncology at West China Hospital of Stomatology, Sichuan University (National Clinical Research Center for Oral Diseases). The recruitment period for this study spanned from October 2024 to January 2025. Upon hospital admission, two nursing staff identified patients diagnosed with oral and maxillofacial malignancies through the electronic medical record system. These nurses underwent a 1-week training program conducted by the researcher to ensure the accurate identification of eligible patients and facilitate their effective participation in the study. After providing a brief overview of the study’s objectives and procedures, the nurse referred eligible patients to the research team. If the patient expressed interest in participation, the research team provided a comprehensive explanation of the study, followed by a thorough eligibility assessment to ensure that all inclusion criteria were met. Regarding caregiver identification, the patient nominated their primary caregiver for participation in the interview. Additionally, contact information, including the patient’s or caregiver’s phone number, was recorded for ongoing communication and supplementary information throughout the study.

The inclusion criteria for patients are as follows: (1) a confirmed diagnosis of oral and maxillofacial malignant tumors, (2) voluntary participation in the study with signed informed consent, and (3) adequate communication skills, facilitating effective participation in interviews. The inclusion criteria for caregivers are as follows: (1) cohabiting with the patient for an extended period and acting as the primary caregiver, (2) voluntary participation in the study, and (3) adequate communication skills, facilitating effective participation in interviews. Exclusion criteria for patients and caregivers include the following: (1) a diagnosis of severe psychiatric disorders, (2) significant cognitive impairments or hearing disabilities that prevent effective cooperation with the study. The sample size was determined based on the principle of data saturation.

### Data collection

To ensure consistency in the timing of data collection, preoperative patients were interviewed at admission, and postoperative patients were interviewed approximately 1 day before discharge. No long-term survivors after treatment were included, thereby minimizing variability related to differences in long-term recovery, adaptation, or motivation.

An initial version of the semi-structured interview guide was developed by the research team based on an extensive literature review and expert consultation. Pilot interviews were subsequently conducted with two participants who met the inclusion criteria, and the interview guide was refined in light of the insights gained. The finalized version is presented in Table 1.

**Table 1:** Topic guide

Oral and maxillofacial cancer patients interview guide
• What is your current understanding of pulmonary rehabilitation? Please elaborate on your knowledge of this concept
• If you were to undergo pulmonary rehabilitation, in which areas would you require additional support?
• What formats or methods would you prefer for receiving guidance or information related to pulmonary rehabilitation?
• During the course of pulmonary rehabilitation, in which areas do you encounter difficulties? How do these challenges affect your rehabilitation process?
• What are your expectations and recommendations for the pulmonary rehabilitation nursing intervention program? Do you identify any potential areas for improvement?
Oral and maxillofacial cancer patients’ caregiver interview guide
• What is your understanding of pulmonary rehabilitation? As a caregiver for a patient, please elaborate on your perception of pulmonary rehabilitation
• If the patient were to undergo pulmonary rehabilitation at home, what potential difficulties or challenges do you foresee? How might these impact their rehabilitation progress?
• During the patient’s pulmonary rehabilitation process, what types of support are you typically able to provide as a caregiver?
• What specific assistance do you hope the pulmonary rehabilitation nursing intervention program could offer to both patients and caregivers?
• During the patient’s pulmonary rehabilitation, through which methods would you prefer to receive relevant information?

The first author, an experienced interviewer with a background in oncology nursing and over 2 years of qualitative research experience, conducted all interviews. There were no pre-existing professional or personal relationships between the authors and the participants before the interviews. Participants completed a brief demographic questionnaire before the interviews, which included information on age, gender, professional specialty, occupation, educational level, and health insurance type. The interviews were held in a quiet meeting room within the department, providing an ideal setting for focused discussions. To accommodate participants’ schedules, the interviews were arranged at their convenience. Interviews, conducted in Chinese, lasted between 20 and 40 min. Data saturation was achieved when participants ceased to provide new information. The resulting textual data, along with the participants’ non-verbal behaviors, were organized and subjected to participant verification to confirm their accuracy. In the data presentation, interviewees are identified as “P” for patients and “C” for caregivers.

### Data analysis

Data collection and analysis were conducted concurrently. Immediately after each interview, the first author transcribed the audio recordings verbatim within 24 h and archived the files in collaboration with a research team member. To maintain the scientific integrity of the analysis, thematic analysis was employed. The key steps in this process included the following: (1) defining the research objectives, (2) organizing the data into categories, (3) developing a coding framework, (4) identifying key themes, (5) repeatedly reviewing and validating the textual data, and (6) analyzing the coded data to refine the themes.

The transcripts were subsequently imported into NVivo 14 software for data management and coding [24]. Initial data extraction and synthesis were independently performed by three researchers with expertise in nursing research and qualitative methodologies. During the first phase of coding, two researchers (JYY and LXK) independently reviewed the transcripts line-by-line, identifying meaningful units related to participants’ experiences, attitudes, barriers, and support needs concerning PR. Relevant keywords, phrases, and actions, such as “lack of understanding,” “need for guidance,” and “psychological burden” were extracted and tabulated alongside illustrative participant quotations.

Following independent coding, the researchers compared their findings, grouping related concepts into preliminary categories. Coding discrepancies were resolved through discussion, achieving 100% consensus after iterative refinement. To further enhance rigor, a third researcher independently reviewed a subset of the data to confirm that the codes and emerging categories accurately reflected the original transcripts. The extracted codes were subsequently clustered into broader conceptual categories. Through iterative comparison and synthesis, key themes and subthemes were developed in direct response to the study’s research questions. The thematic framework was reviewed and refined through ongoing discussions among all authors.

In the final phase, the research team collectively reflected on the derived codes and finalized the major themes. The themes were cross-validated against the full dataset to ensure they remained grounded in participants’ narratives. Representative quotations were selected to illustrate each theme. No new themes emerged from the final transcripts, indicating that data saturation had been achieved. Throughout the analysis, credibility, dependability, and confirmability were maintained through independent coding, constant comparison, consensus discussions, and third-party validation. The findings were reported under the COREQ guidelines.

## Result

### Participant characteristics

A total of 14 participants, comprising both patients (*n* = 7) and caregivers (*n* = 7), were interviewed (Table 2). The participants’ ages ranged from 21 to 70 years, with a mean age of 54.25 years. The mean age of the patients was 57.14 years (range 38–70), while the mean age of the caregivers was 50.1 years (range 21–60). In terms of educational attainment, the majority of patients had completed primary or middle school education (71.4%), whereas most caregivers had a higher level of education, with 3 holding a high school degree (42.9%) and 2 having completed college (28.6%). Regarding occupation, the majority of patients were employed as farmers (71.4%), while most caregivers were either retired or unemployed. The distribution of tumor sites among the patients included buccal, floor of mouth, tongue, soft palate, and gingiva. In terms of clinical staging, most patients were diagnosed with early- to mid-stage disease (stages I–III), with only one case presenting as stage IV.

**Table 2:** Demographic characteristics of interviewees (*n* = 14)

Participant type	ID	Gender	Age (years)	Education level	Occupation	Health insurance	Tumor location	Stage	Interview time
Patient	P1	Male	70	Primary school	Farmer	Urban insurance	Buccal (C06.0)	Stage II	Preoperative
Patient	P2	Male	55	Primary school	Farmer	Urban insurance	Floor of mouth (C04.9)	Stage I	Postoperative
Patient	P3	Male	48	College	Worker	Urban insurance	Tongue (C02.9)	Stage II	Postoperative
Patient	P4	Male	58	Primary school	Farmer	Urban insurance	Buccal (C06.0)	Stage II	Preoperative
Patient	P5	Female	59	Primary school	Farmer	Urban insurance	Tongue (C02.9)	Stage I	Preoperative
Patient	P6	Female	70	Middle school	Farmer	Employee insurance	Buccal (C06.0)	Stage I	Preoperative
Patient	P7	Male	38	College	Employee	Employee insurance	Soft palate (C05.1)	Stage II	Postoperative
Caregiver	C1	Female	60	Primary school	Retired	Social security	Floor of mouth (C04.9)	Stage IV	Preoperative
Caregiver	C2	Male	57	Primary school	Farmer	Urban insurance	Floor of mouth (C04.9)	Stage II	Preoperative
Caregiver	C3	Female	41	College	Unemployed	Commercial insurance	Soft palate (C05.1)	Stage III	Preoperative
Caregiver	C4	Female	21	College	Student	Employee insurance	Gingiva (C03.9)	Stage II	Preoperative
Caregiver	C5	Male	58	High school	Worker	Employee insurance	Gingiva (C03.9)	Stage II	Preoperative
Caregiver	C6	Female	52	High school	Unemployed	Urban insurance	Soft palate (C05.1)	Stage I	Postoperative
Caregiver	C7	Female	34	High school	Unemployed	Urban insurance	Gingiva (C03.9)	Stage III	Postoperative

ICD-10 codes for tumor sites were assigned according to the International Statistical Classification of Diseases and Related Health Problems, 10th Revision (C00–C14). *C06.0*, buccal mucosa; *C04.9*, floor of mouth; *C02.9*, tongue; *C05.1*, soft palate; *C03.9*, gingiva. For patient participants, all demographic and clinical data reflect their own characteristics. For caregiver participants, demographic variables (gender, age, education level, and occupation) refer to the caregivers themselves, whereas all disease-related variables (health insurance, tumor location, stage, and interview time) correspond to the clinical information of the patient they cared for

### Thematic structure: major themes and subthemes

**Fig. 1:**
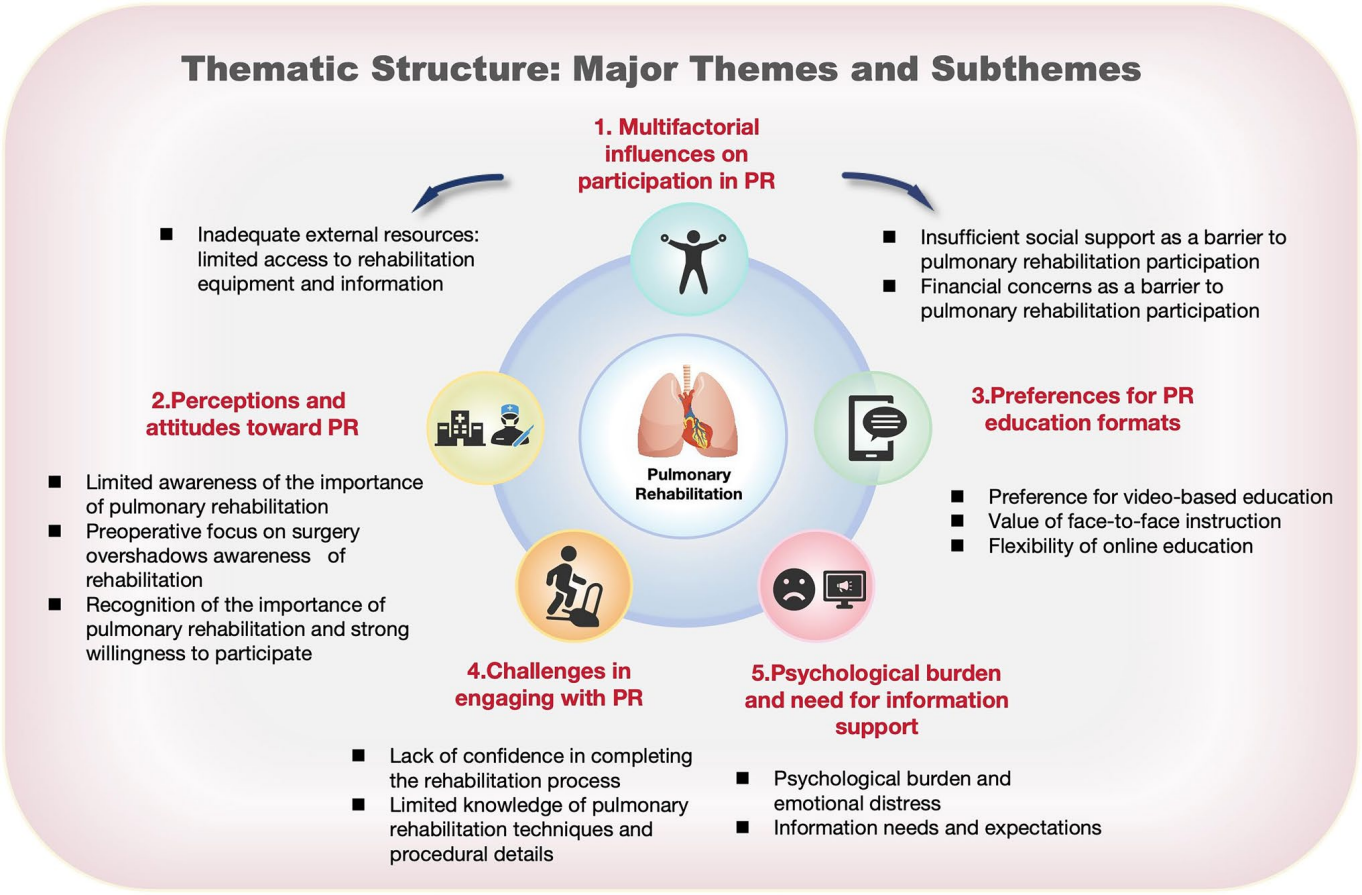
Thematic structure: key themes and subthemes related to pulmonary rehabilitation in oral and maxillofacial cancer patients

Figure 1 presents the five major themes that emerged from the thematic analysis, reflecting the complex experiences, barriers, and needs expressed by oral and maxillofacial cancer patients and their caregivers regarding pulmonary rehabilitation. Each theme is further delineated into subthemes that provide granular insights into participants’ perceptions, challenges, and expectations, offering a nuanced understanding of their lived experiences.

#### Theme 1: Perceptions and attitudes toward pulmonary rehabilitation

### Subtheme 1: Limited awareness of the importance of pulmonary rehabilitation

Some participants, particularly those from rural areas, perceived their daily physical labor as sufficient exercise and thus lacked awareness of the need for structured pulmonary rehabilitation. This misconception appears to stem from limited knowledge of professional rehabilitation practices, leading to an underestimation of PR’s critical role in postoperative functional recovery.

One participant, reflecting on their physical activity, explained: “As a farmer, my day mostly revolves around working in the fields, so I hardly find time for any specific workouts or fun activities. Unless I’m really feeling some discomfort in my body, I usually don’t think too much about other health stuff.” (P6).

Some participants acknowledged that clearer communication from healthcare professionals might enhance their understanding and motivation to engage in PR: “Honestly, I sometimes feel like it doesn’t matter whether I do pulmonary rehab or not. I might try it once or twice and then give up. I didn’t realize how important it is. If you want us to participate, you need to make it clear how important it is, then we’ll take it seriously.” (P2).

For others, the physical demands of daily life diminished their willingness to allocate additional time for rehabilitation: “People usually don’t try to solve a problem until it actually happens. No one thinks about prevention. So, I believe it’s only when complications like those lung problems you mentioned after surgery occur that people start thinking about how to address them.” (C7).

### Subtheme 2: Preoperative focus on surgery overshadows awareness of rehabilitation

During the preoperative period, patients were primarily concerned with the surgical procedure and its success, often neglecting the potential benefits of PR. Many perceived surgery as the definitive solution to their condition, leading to a limited appreciation of PR’s role in enhancing recovery. As one participant reflected: “I never thought about it or looked into it. If someone hasn’t had surgery before, they’re unlikely to think of such things.” (P3).

### Subtheme 3: Recognition of the importance of pulmonary rehabilitation and strong willingness to participate

Upon learning about the benefits of PR, most participants expressed strong support for its value in improving surgical outcomes and recovery. Preoperative PR was seen as a way to reduce postoperative discomfort and enhance overall quality of life. As one participant shared: “During this round of treatment, I realized many problems. The process caused me a lot of harm, which made recovery more painful. If something could have been done earlier to reduce the pain afterward, then I think preoperative rehabilitation would have been worth doing.” (P2).

Several participants noted that earlier awareness of possible pulmonary complications could have motivated greater engagement with PR: “After surgery, I was very weak. When I had to use a ventilator, I couldn’t match its rhythm…if they teach me how to cough up phlegm after the operation, I might be unconscious or in such a bad state that I won’t be able to take it in.” (P3).

Additionally, both patients and caregivers emphasized the need for preoperative health education to enhance preparedness and adherence. One caregiver pointed out: “If they had already practiced certain breathing exercises…doing these exercises in advance would help them improve their recovery.” (C5).

#### Theme 2: Multifactorial influences on participation in pulmonary rehabilitation

### Subtheme 1: Inadequate external resources: limited access to rehabilitation equipment and information

Limited access to rehabilitation equipment and educational materials was a common barrier, particularly among participants from rural areas. Financial constraints and environmental conditions often prevented the acquisition or use of basic PR tools at home. For instance, one participant expressed: “I don’t think most households can have professional rehabilitation machines. But if you could give us some simple tools, like even a balloon, that would be helpful.” (P3). While one participant noted: “I don't understand this professional knowledge because I’ve never had the opportunity to be exposed to it.”(P7).

Inadequate health education formats also impeded engagement. Older adults reported difficulties with visual materials due to small font size or unclear design: “I can’t understand some of the current videos because the font is too small. I can’t read it clearly with my eyesight.” (P4).

Participants further emphasized the lack of accessible resources in their communities. As one explained: “I live in the countryside…there’s no equipment or exercise machines here—they’re only in the cities. But moving to the city isn’t realistic…Plus, I work long hours every day. There’s just no time left for exercise or rehabilitation” (P1).

### Subtheme 2: Insufficient social support as a barrier to pulmonary rehabilitation participation

Family and community support played a critical role in patients’ ability to adhere to pulmonary rehabilitation. Some participants cited the absence of available caregivers to assist with or encourage rehabilitation exercises: “If these exercises require help from someone else, it would be hard…I’m still in school, and other family members are busy too. She can’t complete the exercises on her own” (C1).

Environmental and interpersonal factors further hindered recovery. One caregiver commented: “He’s often exposed to second-hand smoke at home, and the environment isn’t ideal…There’s also a generational gap between us that makes communication harder” (C7).

Concerns about returning to poorly resourced communities after discharge were also expressed: “Our local medical facilities are poor…I’m worried that if we go home, the conditions won’t be good enough for proper recovery” (C6).

### Subtheme 3: Financial concerns as a barrier to pulmonary rehabilitation participation

Economic burden emerged as a significant deterrent to PR participation. While some participants expressed enthusiasm for rehabilitation, they were uncertain about whether they could afford it: “Will there be a fee for this program? If it’s free, I think it would be great.” (C1).

Others echoed the same concern, noting a willingness to engage in PR if the cost was not prohibitive: “I definitely think rehabilitation exercises are necessary. I’m just not sure about the cost. Honestly, I think it’s good to do, whether it’s before or after surgery, as long as it helps. I’d be willing to continue with rehabilitation even after being discharged.” (C3).

#### Theme 3: Preferences for pulmonary rehabilitation education formats

Participants expressed a clear preference for diverse and accessible formats for pulmonary rehabilitation education, with an emphasis on adapting materials to suit varying age groups, cultural backgrounds, and living environments.

### Subtheme 1: Preference for video-based education

Older participants, particularly those with limited literacy or impaired vision, highlighted challenges with written materials. They expressed a strong preference for video-based education, which was perceived as more intuitive and accessible. One participant stated: “It would be great if there were video explanations. Handouts are inconvenient—many older people like me can’t read well, so we don’t understand written materials. Videos have sound and are easier to follow.” (P1).

Younger and middle-aged participants also favored short, video formats for delivering PR information. One participant suggested: “Maybe you could post something on Douyin (the Chinese counterpart of TikTok, widely used for short-form videos in China) and send it to me—that would be helpful, especially for older people. For younger patients like us, a short video or a promotional clip would work too.” (P3).

### Subtheme 2: Value of face-to-face instruction

For participants from rural areas, in-person demonstrations were especially valued. Many felt that face-to-face instruction ensured more accurate execution of rehabilitation exercises. One rural participant emphasized: “If you teach me in person, that would be more effective. I’m from a rural area and used to doing physical work, so I already know how to do some basic exercises. If you demonstrate it on-site, I can pick it up quickly.” (P4).

### Subtheme 3: Flexibility of online education

Participants also expressed a desire for greater flexibility in online education formats. The ability to review instructional content at their convenience was highlighted as a key factor for improving rehabilitation adherence. One participant mentioned: “Sending it to my phone is really helpful. I can watch it during my break in the morning. I’m afraid I’ll forget what you tell me, but if I have the video, I can just check it again anytime.” (P5).

#### Theme 4 Challenges in engaging with pulmonary rehabilitation

### Subtheme 1: Lack of confidence in completing the rehabilitation process

Patients and caregivers frequently expressed uncertainty about their ability to adhere to pulmonary rehabilitation due to physical fatigue, perceived difficulty of the exercises, and doubts about their endurance. Postoperative weakness and the sustained effort required for PR contributed to psychological hesitation.

One patient remarked: “The difficulty is that I don’t know whether I can stick with it, whether I have the confidence to go through the whole pulmonary rehabilitation process from start to finish. I’m not sure I have that kind of determination.” (P6).

Caregivers also noted that some patients lacked motivation or found the exercises too demanding. As one caregiver observed: “She’s a bit lazy, so it’s hard for her to stick with it. Also, it depends on whether the exercises are too difficult—if they’re too challenging, she might not be able to do them.” (C1).

### Subtheme 2: Limited knowledge of pulmonary rehabilitation techniques and procedural details

Participants reported insufficient understanding of specific PR techniques, such as how to pace exercises or execute movements correctly. This lack of procedural clarity often led to confusion and reduced adherence. For example, one patient explained: “It’s hard to get the rhythm right when doing exercises. For example, blowing into a balloon sounds simple, but I don’t know how to control the pace or when to stop. I think if you could make a video, that would make it more intuitive for us.” (P3).

Another added: “I’m not a professional…if you just tell me directly what to do, I’ll go home and follow that. I’ll do as much as I can, whatever step I can manage, I’ll just do that.” (P1).

Despite these knowledge gaps, participants generally expressed trust in healthcare professionals and emphasized the need for individualized instruction. As one participant stated: “I don’t understand this stuff myself, so of course I have no objections to what you suggest. I trust you completely, and I’ll just follow along with whatever pulmonary rehab you tell me to do.” (P6).

#### Theme 5: Psychological burden and need for information support

### Subtheme 1: Psychological burden and emotional distress

Participants reported significant emotional distress related to their illness, particularly during the preoperative phase. Anxiety and fear regarding the surgical procedure and its potential risks were common sources of distress. Such psychological strain may also influence patients’ perceived ability to engage in rehabilitation activities, with several individuals noting that heightened anxiety diminished their confidence, motivation, and readiness to participate in exercises or preoperative preparation. As one caregiver observed: “The surgery is scheduled for tomorrow, and it’s a major one. The patient is anxious. I think it would have helped if there had been some psychological counseling beforehand.” (C1).

Another caregiver expressed anxiety regarding potential complications: “As family members, our biggest concern is how well the patient recovers postoperatively. We worry about complications such as lung infections.” (C2).

This emotional strain was compounded by limited disease understanding and disruptions in social roles after diagnosis. Many participants expressed feelings of inferiority and stigma, leading to social withdrawal and emotional isolation. A caregiver highlighted: “Since he became ill, I feel he’s been experiencing a lot of psychological issues. Being diagnosed has made him feel inferior, and there’s a strong sense of stigma. The emotional burden is quite heavy. If psychological support could be provided, I believe it would help.” (C4).

### Subtheme 2: Information needs and expectations

Participants strongly needed detailed and accessible information about their condition, treatment options, and recovery process. This was especially important regarding potential postoperative complications and rehabilitation expectations. One caregiver suggested: “It would help if patients could be informed about the surgical process ahead of time, so they have a clearer idea of what to expect after the operation and can mentally prepare for it.”(C6).

In addition, patients emphasized the importance of receiving more detailed, tailored health education from healthcare professionals to support informed decision-making and enhance their understanding of their condition. One patient shared: “From a patient’s perspective, I think it would help if healthcare providers explained the disease in more detail. If we understood what’s going on, we wouldn’t feel so afraid or anxious.” (C5).

## Discussion

This qualitative study explored the attitudes, challenges, and support needs associated with pulmonary rehabilitation among patients with oral and maxillofacial cancer and their caregivers in China. Our in-depth qualitative analysis underscores the substantial psychological and systemic barriers that impede engagement with PR. While most participants expressed a general willingness to engage in PR, they also encountered several obstacles, including inadequate access to rehabilitation resources, limited social support, and financial constraints. These findings emphasize the complex interplay of psychosocial and environmental factors that influence patients’ participation in PR. Additionally, participants reported considerable psychological distress, reinforcing the need for timely and personalized information support. Furthermore, the physical and psychological sequelae of cancer diagnosis and treatment, including preoperative symptom burden (such as sadness, stigma, and difficulty swallowing), align with previous studies [25, 26], underscoring the need for comprehensive, patient-centered rehabilitation strategies that address both the physical and emotional needs of this population.

Regarding perceptions and attitudes toward PR, our study found that while participants expressed a general willingness to engage in PR, their understanding of its potential benefits was notably limited. This lack of awareness, particularly among individuals from rural areas, can be attributed to insufficient knowledge of disease management and rehabilitation practices, with a predominant focus on the surgical procedure and its expected outcomes. Consistent with previous studies [27], participants reported physical fatigue as a significant barrier to PR participation, as they perceived it as an additional burden on top of their already demanding daily routines. This finding suggests the need for tailored PR programs that minimize time commitments and address concerns about temporary discomfort, which could otherwise deter participation. Moreover, our results emphasize the critical need for early education on the benefits of PR, particularly before surgery, as McCarron et al. [28] note that a lack of awareness significantly diminishes patients’ likelihood of engaging with and adhering to PR interventions. Aligning with previous research, such as Harris et al. [29], motivation was identified as a pivotal factor influencing engagement with PR. Consequently, interventions aimed at enhancing motivation, such as clearly communicating the benefits of PR, including symptom reduction and improved preoperative conditioning, along with the involvement of family members in the rehabilitation process, could significantly improve patient adherence and overall rehabilitation outcomes.

Several factors were identified as barriers to PR participation in this study, including limited access to external resources, inadequate social support, and financial constraints. A key challenge highlighted by participants was the lack of access to essential rehabilitation equipment, particularly in rural areas with limited medical resources. As one participant noted, “There is no exercise equipment in rural areas; the equipment is all in the city,” emphasizing the inequities in access to rehabilitation services. This limitation suggests the need to design PR programs that minimize reliance on professional equipment, incorporating easily accessible exercises, such as deep breathing, balloon-blowing, and pursed-lip breathing exercises, that can be performed in low-resource settings at home [30, 31]. Beyond equipment shortages, many participants, particularly those with lower educational levels, struggled to understand or apply rehabilitation instructions, with several reporting difficulties such as navigating online exercise resources or determining whether they were performing the exercises correctly. Limited health literacy and restricted access to clear, user-friendly PR materials further hindered meaningful engagement, underscoring the need for simplified and culturally appropriate educational resources. Additionally, participants expressed significant psychological impacts exacerbated by complex treatment regimens and changes in physical appearance. These factors often result in emotional distress, body image concerns, and reduced self-esteem [32]. As a caregiver shared, “Being diagnosed has made him feel inferior, and there’s a strong sense of stigma.” These emotional burdens are compounded by the uncertainty of treatment outcomes and the fear of recurrence [33, 34].

In traditional Chinese culture, family plays a central role in decision-making, particularly when patients are unable, unwilling, or intimidated to express their discomfort or needs [35]. Acknowledging the significant role that family plays in patient care and rehabilitation, we integrated caregivers into our research design to obtain a holistic understanding of patients’ experiences and the support necessary for effective PR. By conducting interviews with both patients and their caregivers, this study aimed to capture the multifaceted perspectives on the challenges and needs associated with PR. The findings underscore the pivotal role of family involvement in the PR process, demonstrating that it not only enhances patient motivation and confidence but also significantly improves the overall effectiveness of rehabilitation interventions [36]. Furthermore, financial concerns emerged as a significant barrier to participation in PR, with many participants in this study expressing concerns about the economic burden of both treatment and rehabilitation. Although most residents in China are enrolled in basic medical insurance schemes, the extent of individual coverage varies, and certain rehabilitation-related expenses may fall outside the reimbursable scope, resulting in additional personal costs for some patients. Similar to findings in head and neck cancer research [37] and patients with chronic respiratory conditions [38], economic burdens related to PR services and health insurance coverage were significant obstacles. To alleviate these concerns, PR programs should consider developing cost-effective models and exploring options for subsidies or insurance coverage. Future research should focus on creating affordable, sustainable rehabilitation programs that ensure greater accessibility and improve patient outcomes, particularly for low-income and rural populations.

This study also highlighted the multifactorial barriers to PR participation among patients with oral and maxillofacial cancer, influenced by both personal and environmental factors. One significant concern was a preconceived lack of confidence in completing the rehabilitation process, primarily due to perceptions of the process as challenging and time-consuming, coupled with an underestimation of their physical capabilities [39]. This lack of confidence was exacerbated by a limited understanding of the specific techniques and procedures involved in PR. These findings align with self-efficacy theory [40], which posits that an individual’s belief in their ability to perform a task significantly influences engagement and persistence. Integrating these principles into PR program design may enhance patients’ confidence, improve adherence, and ultimately lead to better clinical outcomes. Additionally, our study also indicates that the physical and cognitive challenges encountered by patients are often exacerbated by insufficient knowledge support. The absence of skilled healthcare professionals in delivering PR further impedes the effective implementation of rehabilitation programs. To address these challenges, rehabilitation programs should not only provide basic education but also integrate physical activity, nutrition, and psychological support. Nurses, as primary educators, should provide continuous education throughout the perioperative period and during home-based rehabilitation [41], fostering patient engagement and promoting adherence to PR.

This study also integrated findings from Theme 3 and Theme 5, suggesting that several participants experienced heightened psychological burden and emotional distress during the preoperative phase of oral and maxillofacial cancer. However, such experiences likely vary according to disease stage, treatment intensity, and individual circumstances. Rather than indicating a uniform pattern of distress, these findings reflect the lived experiences of the individuals interviewed. Additionally, social role changes and increased family burdens due to lost labor capacity may further exacerbate the psychological strain on both patients and caregivers [42]. As one caregiver noted, “He has become inferior after the disease and hopes to receive psychological support,” indicating that the patient himself experienced diminished self-esteem. These findings reinforce the importance of conducting comprehensive psychological assessments prior to initiating PR to ensure that interventions are appropriately tailored. A tiered approach is essential to address patients’ psychological symptoms, incorporating interventions such as peer counseling and online support groups (which facilitate emotional support and information exchange through digital platforms) [43], and mindfulness techniques may prove beneficial. Previous research supports the effectiveness of mindfulness-based stress reduction, aerobic exercise, and Tai Chi in alleviating psychological symptoms and enhancing resilience during cancer treatment [44] [45]. Furthermore, participants expressed diverse informational needs, particularly regarding treatment options, potential complications, and expected rehabilitation outcomes. Many patients favored a hybrid approach to PR education, combining online and offline resources to accommodate various cultural and environmental contexts. For elderly patients, who often face challenges related to low literacy or impaired vision, traditional written materials were less effective. More accessible formats, such as large-font printed materials, audiobooks, and visually intuitive videos, should be considered. Conversely, younger and middle-aged patients preferred short videos (e.g., Douyin, China’s popular short video app) as an efficient means for quickly understanding rehabilitation content [46]. Tailoring educational materials to meet the specific needs and preferences of different patient groups will not only enhance engagement but also improve the accessibility and effectiveness of PR programs.

The current study possesses several notable strengths and limitations. First, it employs a rigorous design and is the first to comprehensively explore both oral and maxillofacial cancer patients and their caregivers, providing valuable insights into their unique needs and barriers regarding pulmonary rehabilitation. Additionally, the interview schedule demonstrated good face validity, as evidenced by the active engagement of participants in the discussions. Moreover, given China’s rapidly aging population, the study’s focus on pulmonary rehabilitation aligns with national healthcare priorities, particularly the growing emphasis on preoperative rehabilitation as part of current policy initiatives. Nevertheless, there are some limitations in this study. First, the study is limited by its relatively small number of participants. While qualitative research prioritizes depth of insight over statistical representativeness, the modest sample size, especially within the context of China’s large and highly diverse patient population, may restrict the range of perspectives captured and warrants cautious interpretation of the findings. As such, the findings should be interpreted as preliminary, and the study may be more appropriately regarded as a pilot exploration of PR attitudes and needs. Second, all participants were recruited from a single tertiary hospital in China, which may limit the generalizability of the findings to broader populations or varied healthcare contexts. Future studies should consider multi-center recruitment across diverse geographic regions and socioeconomic backgrounds to strengthen the external validity and applicability of the results. Third, while this study offers valuable insights into the experiences of patients and caregivers regarding PR, it did not include the perspectives of healthcare professionals. Incorporating the views of multidisciplinary providers in future research could yield a more comprehensive understanding of PR. Finally, because participants were interviewed only in the immediate pre‑ and postoperative periods, the study lacks longitudinal follow‑up. Future investigations should employ longitudinal designs to capture the dynamic nature of patients’ rehabilitation needs.

## Conclusion and implications for clinical practice

This study is the first to comprehensively identify the multifaceted care needs and challenges associated with pulmonary rehabilitation for patients with oral and maxillofacial cancers and their caregivers in China. Through an in-depth qualitative analysis, the findings underscore the critical need for early education to enhance awareness and encourage participation in PR programs. Oncology healthcare professionals should focus not only on improving patients’ knowledge and skills but also on providing robust psychological support through a variety of approaches within PR programs. Additionally, fostering inclusive practices and creating supportive environments are essential for ensuring the effective delivery of PR. These recommendations could substantially improve patient outcomes, enhance adherence to rehabilitation, and facilitate better recovery, particularly for this vulnerable population. The insights gained from this study can inform the development of culturally tailored, patient-centered PR strategies, thereby contributing to the advancement of healthcare practices in China and other similar contexts globally.

## Supplementary Information

Below is the link to the electronic supplementary material.

ESM1 (DOCX 29.6 KB)

## Data Availability

The datasets generated during and/or analysed during the current study are available from the corresponding author on reasonable request.
