# Supplementary material for: Living with knowledge gap and psychological burdens: understanding the attitudes, barriers, and support needs for pulmonary rehabilitation among oral and maxillofacial cancer patients in China
Source: Support Care Cancer. 2026 Feb 6;34(3):170. doi: 10.1007/s00520-025-10301-6 (PMC12881002; doi:10.1007/s00520-025-10301-6)
Supplement: Supplementary file 1 — (DOCX 29.6 KB) [file 520_2025_10301_MOESM1_ESM.docx]

**Table: Thematic structure: key themes and subthemes related to pulmonary rehabilitation in oral and maxillofacial cancer patients**

| **Theme** | **Subtheme** |
| --- | --- |
| **Perceptions and attitudes toward PR** | Limited Awareness of the Importance of Pulmonary Rehabilitation |
|  | Preoperative Focus on Surgery Overshadows Awareness of Rehabilitation |
|  | Recognition of the Importance of Pulmonary Rehabilitation and Strong Willingness to Participate |
| **Multifactorial influences on participation in PR** | Inadequate External Resources: Limited Access to Rehabilitation Equipment and Information |
|  | Insufficient Social Support as a Barrier to Pulmonary Rehabilitation Participation |
|  | Financial Concerns as a Barrier to Pulmonary Rehabilitation Participation |
| **Preferences for PR education formats** | Preferences for Pulmonary Rehabilitation Education Formats: Integration of Online and In-Person Delivery with Personalized Needs |
| **Challenges in engaging with PR** | Lack of Confidence in Completing the Rehabilitation Process |
|  | Limited Knowledge of Pulmonary Rehabilitation Techniques and Procedural Details |
| **Psychological burden and need for information support** | Psychological Burden and Emotional Distress |
|  | Information Needs and Expectations |

| **Table. Patient Interview Summary on pulmonary rehabilitation** | | | | | | | |
| --- | --- | --- | --- | --- | --- | --- | --- |
| **Interviewee** | **Patient 1** | **Patient 2** | **Patient 3** | **Patient 4** | **Patient 5** | **Patient 6** | **Patient 7** |
| **Interview outline** | | | | | | | |
| **1.What is your current understanding of pulmonary rehabilitation? Please elaborate on your knowledge of this concept.** | I had never heard of it before, but after your introduction, I believe it can help us prepare for surgery in advance and approach it in a better physical state. | I haven't researched it, but through this treatment, I realized that many things still caused me harm during the process, making the later recovery quite painful. If it could have been improved earlier, it would have reduced the pain in the recovery stage. I think it would still be worthwhile to consider this before surgery. | I had neither considered nor researched it before, as I never thought about it before undergoing surgery. However, I now believe it is necessary. After the surgery, I felt very weak, and if I had needed a ventilator, I would have had trouble keeping up with its rhythm. It is difficult to control the breathing rhythm, which could be a problem. Also, if they teach me how to cough up phlegm after surgery, but if I’m in a coma or in poor condition post-surgery, I wouldn’t be able to listen or could feel helpless. If I had been informed in advance, I would have been more aware of how to handle it. | I haven’t looked into it, but I think it is quite important, as exercise is beneficial for the body. I see it as an extension of my life. | I haven't explored it before, but I believe rehabilitation is necessary. If the lung capacity increases, the body should improve as well. | My brother has undergone heart surgery. He mentioned that after the surgery, he did exercises at home, like blowing balloons and doing breathing exercises. I think that approach works. However, for me, it might not be very important. If I suddenly remember, I can do it, but when busy, I won’t think about doing such exercises. My health is generally good; I rarely even catch a cold. | I’ve had surgery once, and now I’m undergoing a second one. I’m generally familiar with the process. I had a CT scan yesterday, and the doctor mentioned there was still some inflammation in my lungs. My son often tells me to exercise regularly, and I do exercise at home. I believe regular exercise is still good for the body. |
| **2.If you were to undergo pulmonary rehabilitation, in which areas would you require additional support?** | I am not an expert in this area, and I don’t quite understand it. I believe you just need to tell me face-to-face what I need to do, and I can do it at home. I will do as much as I can, step by step, because I work on the farm and often get physical exercise. | Actually, the key is in the method. I think as long as the instructions are clear to the patient, it should be fine. If you don’t explain exactly how to do it, I will just implement it based on my own understanding. However, if you tell me specific times, quantities, and frequencies, I might be able to follow through more effectively. | I believe that at home, you can’t really equip me with machines, but simple tools, like giving me a balloon, would be good. However, I don’t know exactly how to blow it up—like whether to blow three times and pause for two, or how to manage the rhythm. I think there should be more guidance on this aspect. | I think you need to explain how to operate it, as I don’t understand anything at all about it, including disease knowledge and how to exercise. I know nothing. | I hope the content is simpler so that it's easier to follow. If there were videos, it would be even better, as videos are very visual, and they often include text. I could watch them on my phone, and whenever I want, I can view them wherever I am. A simple face-to-face demonstration of the exercises would also work. | I actually don’t have these habits. We are farmers, and we work in the fields every day. Unless there is a problem, I don’t worry about other things. Life is hard, and we have no choice but to work. There’s so much farm work that I don’t even participate in square dancing. We don’t have any entertainment. After finishing the work, I rest in the evening and play on my phone. | I don’t really understand this professional knowledge because I’ve never had the opportunity to be exposed to it. |
| 1. **What formats or methods would you prefer for receiving guidance or information related to pulmonary rehabilitation?** | It’s more intuitive if you explain it to me face-to-face. Videos are also fine, but sending a manual would be inconvenient, as many elderly people, including myself, cannot read. We wouldn’t be able to understand the manual, but with videos, we can hear the instructions, which makes it more convenient. | I think videos are better. If the videos include well-structured content that you’ve already prepared, you can send them to us, and we can follow along at home. It’s also important that you explain the exercises clearly to the patients, so we don’t have to figure it out ourselves. The video should clarify every detail. | For example, if you record something on Douyin (a Chinese short-video platform) and send it to me, that might be more suitable for elderly people. For younger people, sending a small advertisement or a promotional video might work. Also, as you mentioned, if you could provide rental machines or equipment during the hospital stay, or set up exercise locations in public places, that would be helpful. I could use them to exercise when I arrive. | Teaching me in person would be the most effective approach, because I have been exercising regularly in the countryside, and I know how to do it. So, if you show me in person, I can quickly learn. | I don’t need the video, just an in-person explanation. I can’t understand the videos, and the text is often too small for me to read. | It would be great if you could send this information to me on my phone. For example, when I’m resting in the morning, I can open my phone to check. I worry that I might forget what you’ve taught me, but if it’s on my phone, I can easily look it up if I forget. | Teaching me face-to-face would be best because I don’t know how to use a phone. If you teach me in person, I can ask questions if I don’t understand something. |
| 1. **During the course of pulmonary rehabilitation, in which areas do you encounter difficulties? How do these challenges affect your rehabilitation process?** | I believe there are still many difficulties because I live in the countryside, where there are no exercise equipment or machines; all those facilities are in the city. Moving to the city isn’t practical either, as I need to take care of elderly family members. Additionally, I drive a truck for work, and my working hours are long. I have little time for exercise, so I can't devote time to these activities. | The only problem is that I may feel that it doesn’t matter whether I do the exercises or not. I might end up doing less or quitting after a couple of days because I don’t realize how important it is. | I find it hard to control the rhythm of exercise. For example, when you ask me to blow up a balloon, it seems simple, but I’m unsure how to maintain the right rhythm or when to stop.If we could record a video, I think it would be more intuitive to follow along. | There shouldn’t be much difficulty. | I should say there are no difficulties. I walk several kilometers every day and have been consistently exercising.， | The difficulty, however, is not knowing if I can persist. I’m uncertain whether I have the confidence to complete the entire exercise regimen from start to finish. | As long as you tell me how to do it, I will follow through. I don’t think it will be a problem. I believe I can stick to it, especially if it’s good for my health. I have no problem with activities like climbing stairs in my daily life. |
| **5.What are your expectations and recommendations for the pulmonary rehabilitation nursing intervention program? Do you identify any potential areas for improvement?** | No issues. | I previously participated in a follow-up program at another hospital, but after paying the fee, no one contacted me again. Even when my symptoms became severe, I received no support. I hope that staff can maintain timely communication with patients, as this helps us provide feedback. Moreover, when involving patients in training, it's important to let them understand its significance; only then will they be motivated to participate. | Ideally, the simpler the approach, the better. For example, if I am waiting for surgery for ten days, it would help to know what to do during the first two days and what to do during the last two days. | I believe exercise is about being responsible for one's own body. With this mindset, I can stay committed. As long as it benefits my health, I am willing to do it. | No issues. | I don’t understand these things myself, so I certainly have no complaints about your work. I trust you and will simply follow your instructions. | I hope the delivery methods can be diverse. |

| **Table Caregivers Interview Summary on pulmonary rehabilitation** | | | | | | | |
| --- | --- | --- | --- | --- | --- | --- | --- |
| **Interviewee** | **Patient 1** | **Patient 2** | **Patient 3** | **Patient 4** | **Patient 5** | **Patient 6** | **Patient 7** |
| **Interview outline** | | | | | | | |
| **1.What is your understanding of pulmonary rehabilitation? As a caregiver for a patient, please elaborate on your perception of pulmonary rehabilitation.** | I hadn’t heard of this before, but after your explanation, I feel that pulmonary rehabilitation is quite beneficial. If it helps improve health, we are willing to accept it. Does this program require payment? If it’s free of charge, I think it’s excellent. | I’m not familiar with it. But if it can help improve lung function, I think it’s great, as it may assist in recovery after surgery. As family members, our biggest concern is how well the patient recovers postoperatively. We worry about complications such as postoperative pulmonary infections. | I believe rehabilitation exercises are necessary, as they might significantly reduce negative outcomes after surgery, right? Although we’ve heard of similar rehabilitation programs, we haven’t personally experienced them. Still, I believe they are meaningful for patients. I’m also unsure about the cost. Overall, I think this is great. Whether before or after surgery, anything that’s beneficial should be done. We would also be willing to participate in rehabilitation programs after discharge. | I haven’t heard of this before. Typically, people are most concerned with surgical details and not much else. | After patients return to the ward, they may face issues like swallowing difficulties due to lack of training or knowledge. Undergoing this surgery is quite painful. If there are methods available before surgery that help patients become aware of what to expect—for instance, specific breathing exercises—then they could be used when symptoms arise. I think this is necessary. During the waiting period at home, patients often have nothing to do, so starting these exercises early could help them prepare for further rehabilitation. Perhaps we’ve had very little exposure to this illness. We only found out yesterday how painful it can be. We initially thought it was a simple issue, but it turned out not to be. I’ve only seen one case so far, so I had never considered these problems. As family members, we didn’t know postoperative respiratory complications could arise. For example, I saw the patient in the next bed struggling with swallowing and discomfort after surgery. | I hadn’t heard of it before, but after surgery, he had a lot of phlegm, and the nurse would come to suction it, and he would cough frequently. Based on what you’ve just described, I think pulmonary rehabilitation and exercises are very beneficial. I have limited education, so we simply follow the doctor’s instructions. As long as the benefits are explained clearly, we will follow through, especially if it aids recovery. | I wasn’t aware of this because he didn’t have any preexisting lung conditions, so I don’t know much about it. After the surgery, however, he did produce a lot of saliva. Pulmonary rehabilitation, as you explained, is about exercising before surgery to improve lung function, which can promote faster recovery. |
| **2.If the patient were to undergo pulmonary rehabilitation at home, what potential difficulties or challenges do you foresee? How might these impact their rehabilitation progress?** | She is too lazy, she won’t be able to stick to the exercises. Additionally, it depends on the difficulty of the exercises; if they are too challenging, she might not be able to do them. Also, if the exercises require assistance from others, it may not work, because I am in school and others do not have time to help. There is no one at home to assist her, so she may not be able to complete them on her own. | I think he is lacking self-discipline, and he will not able to perform the exercises well because I am in school, and I feel I won’t be able to supervise and guide him effectively. | There shouldn’t be too many difficulties. After the healthcare staff provides guidance on what exercises to do, family members will certainly supervise them to ensure they are completed. Family members are usually very willing to monitor their progress. | I can’t think of any potential difficulties at the moment. I think whatever you tell us to do, we will just follow your instructions. We generally listen to doctors and nurses. | Because we didn’t know much about this disease, we thought it was very simple when we first encountered it. We also thought it was a very minor surgery, and we would be back home in a day or two. So we were not mentally prepared at all. As a result, we found out that this surgery was not simple at all after we came in. Everyone was a little anxious and might be afraid to do it. I should have let the patient know what the risks of this surgery were in advance, and then he could consult with all parties to understand it, so that he would not be so panicked when he came. Otherwise, I don’t know anything, and I am really afraid of surgery. | The difficulties should be minimal. He will still cooperate well. | I think he will be exposed to secondhand smoke quite often, as the environment is not ideal, which may not be conducive to recovery. When guiding him in exercises, it depends on whether he listens to advice. I am concerned that he might not want to do them, as there seems to be a generation gap in terms of cognition between him and us, the children. Generally speaking, people tend to address problems only after they arise, rather than proactively thinking of solutions. So, I believe that only after encountering issues like the pulmonary complications you mentioned will he begin to think about how to resolve them. |
| **3.During the patient's pulmonary rehabilitation process, what types of support are you typically able to provide as a caregiver?** | As mentioned earlier, I would prefer face-to-face guidance, as I don’t fully understand many aspects. | Whatever the medical staff believes the family members need to do, just let us know directly—we will fully cooperate. | The main issue is that we don’t understand this disease or the relevant medical knowledge. We’re unfamiliar with how it works. If disease education could be provided, that would be very helpful.。 | I hope psychological support can also be offered. | I think the main support I can offer is emotional—encouraging him and ensuring he attends necessary check-ups. Right now, I hope he can be discharged later rather than sooner. The medical resources in our hometown are inadequate, and this surgery was quite major. I’m concerned that the recovery may not go well at home. Since we’re from Yunnan, coming back to the hospital would be difficult due to the distance. We’ve already been here for a month. | I think the main support I can offer is emotional—encouraging him and ensuring he attends necessary check-ups. Right now, I hope he can be discharged later rather than sooner. The medical resources in our hometown are inadequate, and this surgery was quite major. I’m concerned that the recovery may not go well at home. Since we’re from Yunnan, coming back to the hospital would be difficult due to the distance. We’ve already been here for a month. | All I can do is try to explain the benefits of pulmonary rehabilitation and how it can improve his condition, and encourage him to exercise as much as possible.。 |
| **4.What specific assistance do you hope the pulmonary rehabilitation nursing intervention program could offer to both patients and caregivers?** | Will there be postoperative follow-ups? I believe follow-up is very important, especially to inquire about our recovery status. The surgery is scheduled for tomorrow, and it’s a major operation. The patient is very anxious. It would be helpful if some psychological counseling were provided beforehand. | What we need most is knowledge-based guidance, because we don’t understand anything about this disease or the surgery, let alone the rehabilitation process. Since we haven’t undergone surgery yet, we know even less about what rehabilitation entails. | I’m not clear on the specific content, since pulmonary rehabilitation involves professional knowledge, which we don’t really understand. | After falling ill, I think he developed many psychological issues. He feels ashamed and inferior about his condition, and he carries a heavy mental burden. If your videos could also provide some form of psychological support, I think that would be very beneficial. | I think you could create something to help us understand the symptoms that may arise after surgery, so that we aren’t overwhelmed when they occur. It would also help to explain the surgical process to the patient in advance, so they have a clearer understanding of what to expect after surgery. This would help them prepare both mentally and practically. For instance, you could offer a Q&A format, where patients ask questions and healthcare providers respond—explaining what to do if certain issues arise. I believe that if medical staff could describe this illness in detail, especially from their own experience or understanding, patients would better grasp the nature of their condition. | It would be ideal if the patient’s condition could be assessed before surgery. | If the department could directly provide recommendations or a comprehensive plan for the patient to review, he would be more likely to appreciate its importance and be more motivated to stick to the rehabilitation. He places great trust in doctors’ advice. |
| **5.During the patient's pulmonary rehabilitation, through which methods would you prefer to receive relevant information?** | Hands-on, face-to-face demonstrations are very effective; they allow us to learn quickly. | I think face-to-face instruction is great because it makes the explanation clearer and easier for me to understand. | In-person explanations are quite helpful, as many people may not even read the manuals if they’re handed out. | I believe watching videos is very effective since they are more intuitive and easier to understand. For example, if the video explains the benefits of these exercises for the patient, we would be more motivated to stick to them if we see that they’re good for recovery. We are also hoping to have some of our preoperative questions answered, as we don’t fully understand the details of the surgery. | Some patients might not perform the exercises correctly or may lack persistence, which is often related to personal habits. I think you could provide a video. If patients watch it and save it, they can revisit it as needed. Videos are convenient. However, since some people don’t like watching videos and others lack self-discipline, you might also prepare a simple written guide. Everyone has different preferences. Generally, if something concerns one’s own health, they’ll pay attention to it. I think the video should be simple, not complicated—easy to understand, with minimal steps. Most people prefer clear and straightforward “foolproof” methods. If it’s too complicated, people might give up. Additionally, I think you could record a video to address the psychological aspect of the disease. If your staff is short on time, even a single video could be helpful—for instance, showing that the illness isn’t something to fear. This would help reduce patients’ anxiety, inform family members about the condition, and boost patients’ confidence by highlighting the high recovery rate and the manageability of the disease. | I can generally remember what you tell me, but having a video would be better. I might forget things, and if there’s a video, I can watch it again anytime. | I think any format is acceptable, but a handbook might be more helpful for reviewing at home. I also hope you can provide some information about follow-up appointments, such as when we should return for review. |
